# The Second Chromosome Promotes the Adaptation of the Genus *Flammeovirga* to Complex Environments

**DOI:** 10.1128/Spectrum.00980-21

**Published:** 2021-12-08

**Authors:** Zewei Feng, Zheng Zhang, Yu Liu, Jingyan Gu, Yuanyuan Cheng, Wei Hu, Yuezhong Li, Wenjun Han

**Affiliations:** a National Glycoengineering Research Center, Shandong Key Laboratory of Carbohydrate Chemistry and Glycobiology, NMPA Key Laboratory for Quality Research and Evaluation of Carbohydrate-based Medicine, and State Key Laboratory of Microbial Technology, Shandong University, Qingdao, China; b United Post-Graduate Education Base of Shandong University and Jinan Enlighten Biotech, Co., Ltd., Jinan, China; c School of Food Science and Engineering, Shandong Agriculture and Engineering University, Jinan, China; South China Sea Institute of Oceanology, Chinese Academy of Sciences

**Keywords:** evolution, *Flammeovirga*, multiple replicons, polysaccharide degradation, primary chromosome, secondary chromosome

## Abstract

Approximately 10% of bacterial strains contain more than one chromosome; however, in contrast to the primary chromosomes, the mechanisms underlying the formation of the second chromosomes and the significance of their existence remain unclear. Species of the genus Flammeovirga are typical polysaccharide-degrading bacteria, and herein, we report complete genome maps of this genus. These genomes all had multireplicons and second chromosomes. The second chromosome, much larger than plasmids and even megaplasmids, had rRNA and a disparity of 1% relative to the main chromosome in guanine-cytosine (GC) content. The largest chromosomes carried core genes for cellular processes, while the second chromosomes were enriched with genes involved in the transport and metabolism of inorganic ions and carbohydrates, particularly genes encoding glycoside hydrolases and polysaccharide lyases, which constituted the genetic basis for the strains’ excellent capabilities to utilize polysaccharides. The second chromosomal evolution had a higher mutation rate than the primary chromosomes. Furthermore, the second chromosomes were also enriched in horizontal transfer genes and duplicated genes. The primary chromosomes were more evolutionarily conserved, while the second chromosomes were more plastic, which might be related to their different roles in the bacterial survival process. This study can be used as an example to explain possible formation mechanisms and functions of the second chromosomes, providing a reference for peer research on the second chromosomes. In particular, the second chromosomes were enriched in polysaccharide-degrading enzymes, which will provide theoretical support for using genomic data to mine tool-type carbohydrase resources.

**IMPORTANCE** For decades, the typical bacterial genome has been thought to contain a single chromosome and a few small plasmids carrying nonessential genes. However, an increasing number of secondary chromosomes have been identified in various bacteria (e.g., plant symbiotic bacteria and human pathogens). This study reported three complete genomes of the polysaccharide-degrading marine bacterial genus Flammeovirga, revealed that they harbor two chromosomes, and further identified that the presence of a multireplicon system is a characteristic of complete Flammeovirga genomes. These sequences will add to our knowledge on secondary chromosomes, especially within Bacteroidetes. This study indicated that the second chromosomes of the genus Flammeovirga initially originated from an ancestral plasmid and subsequently expanded by gene duplication or by obtaining heterologous genes with functions, thus promoting host strains to adapt to complex living environments (e.g., to degrade more diverse polysaccharides from marine environments). These findings will promote the understanding of the evolution and function of bacteria with multireplicon systems.

## INTRODUCTION

The bacterial genome usually consists of a single circular chromosome; however, recent genomic studies have indicated that approximately 10% of bacterial strains contain more than one chromosome. Similar to the largest (primary) chromosomes, the secondary chromosomes also carry partial core genes essential for bacterial growth, whereas they possess plasmid-type DNA replication and assignment systems (1). Interestingly, secondary chromosomes have mostly been identified in many important bacteria, such as Agrobacterium (2) and Rhizobia (3), which are plant symbiotic bacteria affecting agricultural production, and Brucella (4), Burkholderia (5), and Vibrio (6), which are human or animal pathogens. It is believed that bacterial secondary chromosomes will be identified and reported in increasing numbers with improvements in genome-sequencing technologies. However, relatively little is known about how the second chromosomes were formed and what effects they have on the survival of their host strains.

Most of the species within the genus Flammeovirga, belonging to the family Flammeovirgaceae of the phylum Bacteroidetes (7), are currently identified as efficient polysaccharide-degrading marine bacteria. These strains have been isolated from the deep sea, coastal sediments (7–9), algae surfaces (10), or marine animal coelenterates (11). For example, Flammeovirga sp. MY04, isolated by our group from coastal sediment (7), is a strain enriched in polysaccharide-degrading enzyme-encoding genes. A series of glycosyl hydrolase genes, such as agarose (12, 13), mannose hydrolase, and polysaccharide lyase, such as algin lyase (14–16) and xanthan gum lyase, were cloned and identified from strain MY04; Flammeovirga yaeyamensis strain NBRC 100898 was isolated from *Yaeyama* Island (17) in 2006. Flammeovirga kamogawensis strain YS10 was a strain isolated from seawater off the coast of Japan (8) in 2007, and Flammeovirga pectinis strain L12M1 was isolated from the viscera of Korean scallops in 2018 (18). However, most of these bacteria belonging to the genus Flammeovirga have been reported in the draft genome (19). Due to the lack of characteristic information on the composition, structure, and function of the genomes of this genus, it is very difficult to further understand the ecological function, characteristics, and genetic basis of the strains.

Here, we sequenced, assembled, and annotated the genomes of several typical Flammeovirga strains, including strains MY04, NBRC 100898, and YS10, and found that they all have secondary chromosomes. Therefore, we compared and analyzed the composition, function, and evolutionary characteristics of the genome of all known strains with complete genome information, especially the differences among different replicons. The existence and possible formation mechanisms of the second chromosomes in Flammeovirga genomes and their influences on the environmental adaptabilities of the strains were analyzed and discussed.

## RESULTS

### The genomes of the genus Flammeovirga contain second chromosomes.

**FIG 1:**
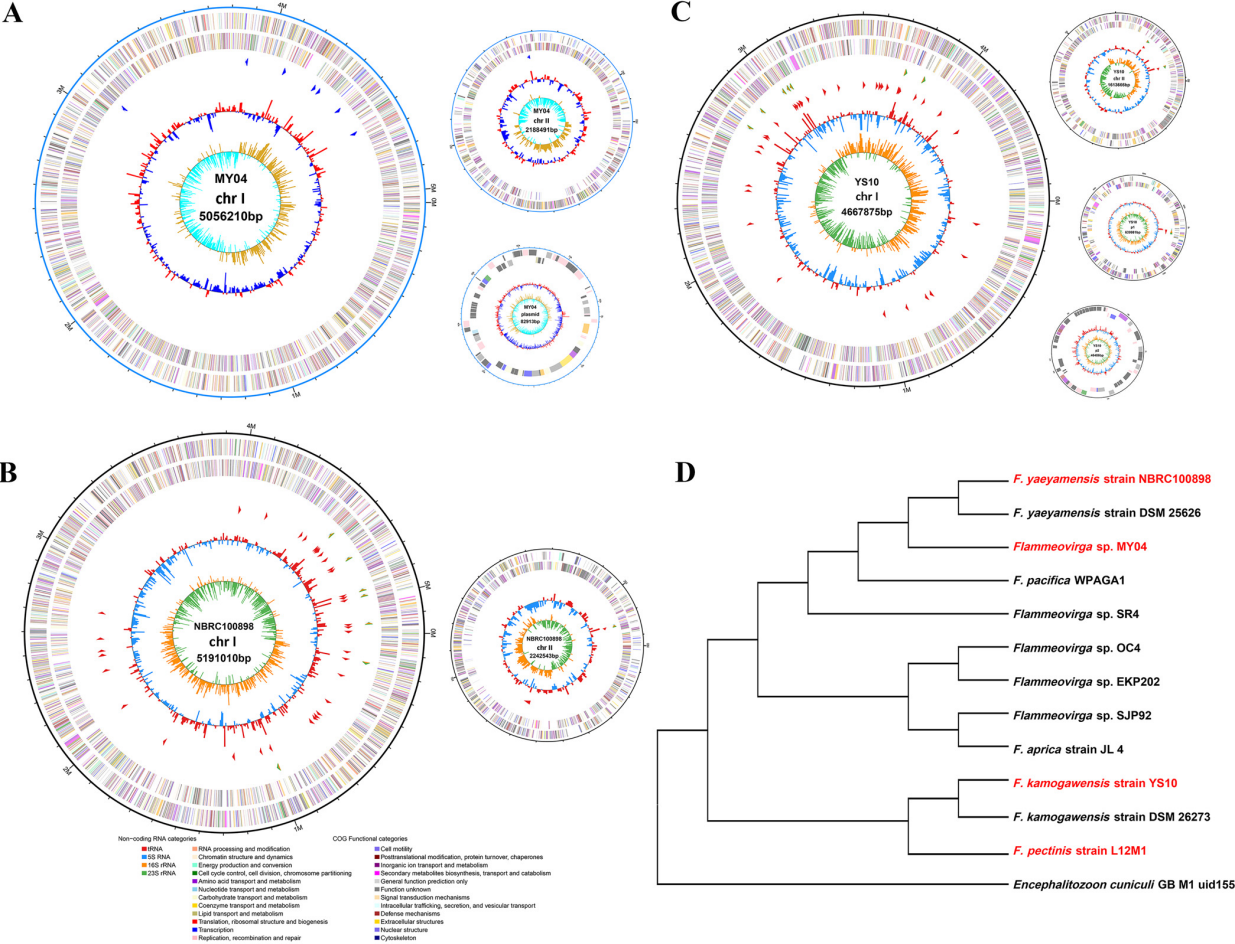
Flammeovirga genome circle maps and the evolutionary tree. (A) MY04. (B) NBRC 100898. (C) YS10. (D) Evolutionary tree. Schematic representation of the chromosomes and plasmids. Radii are scaled based on replicon sizes, except for the plasmids, because the plasmids are so small. The outermost circle of the circle diagram is the identification of the genome size, the second circle and the third circle are the CDSs on the forward and reverse strands, and different colors indicate the functional classification of the cluster of orthologous gene (COG) of the CDS. The fourth circle is the GC content, and the fifth circle is the G+C skew. The evolutionary tree is constructed based on the genome. This indicates the evolutionary status of all Flammeovirga sp. strains with genomic information. Phylogenetic analysis showed that the strain closest to MY04 was NBRC 100898, and the strain closest to YS10 was L12M1.

In 2004, Flammeovirga sp. strain MY04 was isolated from coastal sediment in eastern China. The bacterium showed a curved rod shape, grew well at 30°C and pH 7, and exhibited a significant color change (deep red-orange in the exponential growth phase and a white color in the later stage). Our previous work indicated that strain MY04 was a polysaccharide-degrading bacterium that could grow well on a variety of polysaccharide carbon source-limited media, such as agar, xylan, mannan, cellulose, and carrageenan (7). The complete genome sequence of Flammeovirga sp. MY04 was determined and assembled using Illumina HiSeq and Pacific Biosciences sequencing technologies (RefSeq Assembly Acceptance GCF_001682195.2). The complete genome size of the strain was 7,327,614 bp, encoding 5,618 genes that included 5,495 protein-coding genes, 24 rRNA genes, and 86 tRNA genes (Table 1). The genes for MY04 were distributed across three circular replicons named chromosome I (5.06 Mbp, 4,066 genes), chromosome II (2.19 Mbp, 1,481 genes), and plasmid (0.08 Mbp, 71 genes) (Fig. 1A). We found that the smaller replicon II of MY04 (i) encoded rRNA, while the rRNA (*rrn*) operon that encoded rRNA (16S, 23S, and 5S rRNA) was a marker that defines the replicon as a “chromosome” (20), and (ii) their sizes were between 1.41 Mb and 2.21 Mb, which was much larger than the average size of the plasmid (78.9 kb) and the average size of the megaplasmid (772 kb) (1), and (iii) the difference in GC content between chromosome II and chromosome I was no more than 1% (for example, 34% to 33.5% < 1%) (1). Taken together, we defined the replicon as chromosome II rather than a plasmid or megaplasmid.

**TABLE 1:** General features of the Flammeovirga genomes

Strain	Type	RefSeq	INSDC	Size (Mb)	GC (%)	No. of proteins	rRNA	tRNA	No. of Genes
MY04	Chromosome I	NZ_CP003560.2	CP003560.2	5.06	34.6	3,951	21	85	4,066
	Chromosome II	NZ_CP003561.2	CP003561.2	2.19	34.6	1,474	3	1	1,481
	Plasmid	NZ_CP003562.2	CP003562.2	0.08	31.1	70			71
NBRC 100898	Chromosome I	NZ_CP076132.1	CP076132.1	5.19	34.5	4,002	21	90	4,125
	Chromosome II	NZ_CP076133.1	CP076133.1	2.24	34.6	1,558	3	1	1,565
YS10	Chromosome I	NZ_CP076128.1	CP076128.1	4.67	32	3,666	21	96	3,791
	Chromosome II	NZ_CP076129.1	CP076129.1	1.61	31.4	1,096	6	2	1,106
	Plasmid	NZ_CP076130.1	CP076130.1	0.64	32.4	419	3	1	425
	Plasmid	NZ_CP076131.1	CP076131.1	0.05	31.4	48			49
L12M1	Chromosome I	NZ_CP034562.1	CP034562.1	5.21	32.3	4,014	24	95	4,141
	Chromosome II	NZ_CP034563.1	CP034563.1	1.41	31.8	993	6	3	1,000
	Plasmid	NZ_CP034564.1	CP034564.1	0.06	29.4	56			58

The sequencing and assembly of the genomes of F. yaeyamensis strain NBRC 100898 (RefSeq Assembly Acceptance GCF_018736045.1) and F. kamogawensis strain YS10 (RefSeq Assembly Acceptance GCF_018736065.1) also revealed that they had multiple replicons. The smaller replicon II of these strains, such as replicon II of MY04, had chromosome characteristics, so we named them chromosome II. Among them, the complete genome size of NBRC 100898 was 7,433,553 bp, encoding 5,690 genes that include 5,560 protein-coding genes, 24 rRNA genes, and 91 tRNA genes (Table 1). The genes were distributed on two circular replicons, namely, chromosome I (5.19 Mbp, 4,125 genes) and chromosome II (2.24 Mbp, 1,565 genes) (Fig. 1B). The complete genome of YS10 was 6,968,008 bp in size and encoded 5,371 genes, including 5,229 protein-coding genes, 30 rRNA genes, and 99 tRNA genes (Table 1). The genes were distributed on four circular replicons, namely, chromosome I (4.67 Mbp and 3,791 genes), chromosome II (1.61 Mbp, 1,106 genes), plasmid I (0.64 Mbp, 425 genes), and plasmid II (0.05 Mbp, 50 genes) (Fig. 1C). It has recently been reported that the genes of F. pectinis strain L12M1 (RefSeq Assembly Acceptance GCF_003970675.1) are distributed in three circular replicons, namely, chromosome I (5.21 Mbp, 4,141 genes), chromosome II (1.41 Mbp, 1,000 genes), and plasmid (0.06 Mbp, 58 genes) (Table 1). In short, these three fully sequenced genomes also contain two chromosomes like MY04.

Based on the complete genome sequences, phylogenetic analysis showed that the strain closest to MY04 was NBRC 100898, and the strain closest to YS10 was L12M1. Moreover, the four strains were located in two distant branches in the phylogenetic tree, suggesting that the second chromosomes were a common feature of the genus (Fig. 1D).

In the process of cell growth and division, approximately three-quarters of the bacterial genetic information is segregated by the ParA-ParB-*parS* system, consisting of an ATPase protein ParA, a CTPase and DNA-binding protein ParB, and a centromere-like *parS* site (21). RepA is the replication initiator protein in Pseudoalteromonas chromids (22). The fundamental function of the partitioning system and the replication initiator protein, together with their widespread distribution in bacteria and archaea, make ParA, ParB, and RepA excellent candidates for finding clues to the origin of the second chromosome (23). In MY04, NBRC 100898, YS10, and L12M1, we found that the homologs of *dnaA* and *dnaN*, the key functional genes responsible for chromosome replication, existed only on chromosome I, while the homologs of ParA, ParB, and RepA had copies on chromosome II and the plasmid, respectively. To investigate where the second chromosome originates from, we performed BLASTp searches using the second chromosome ParA, ParB, and RepA proteins as queries against the nr. database. ParA and ParB BLASTp searches identified homologs from draft genomes of Thalassobius sp. and Oxalobacter formigenesas as best hits (e values = 3e−41/8e−19, identities = 33.57%/29.07%), respectively. RepA BLASTp searches identified homologs from draft genomes of Sediminitomix flava as best hits (e values = 2e−28, identities = 26.65%), suggesting that replicon II was likely to come from an early plasmid.

### There were functional differences between the two chromosomal coding genes of the genus Flammeovirga.

**FIG 2:**
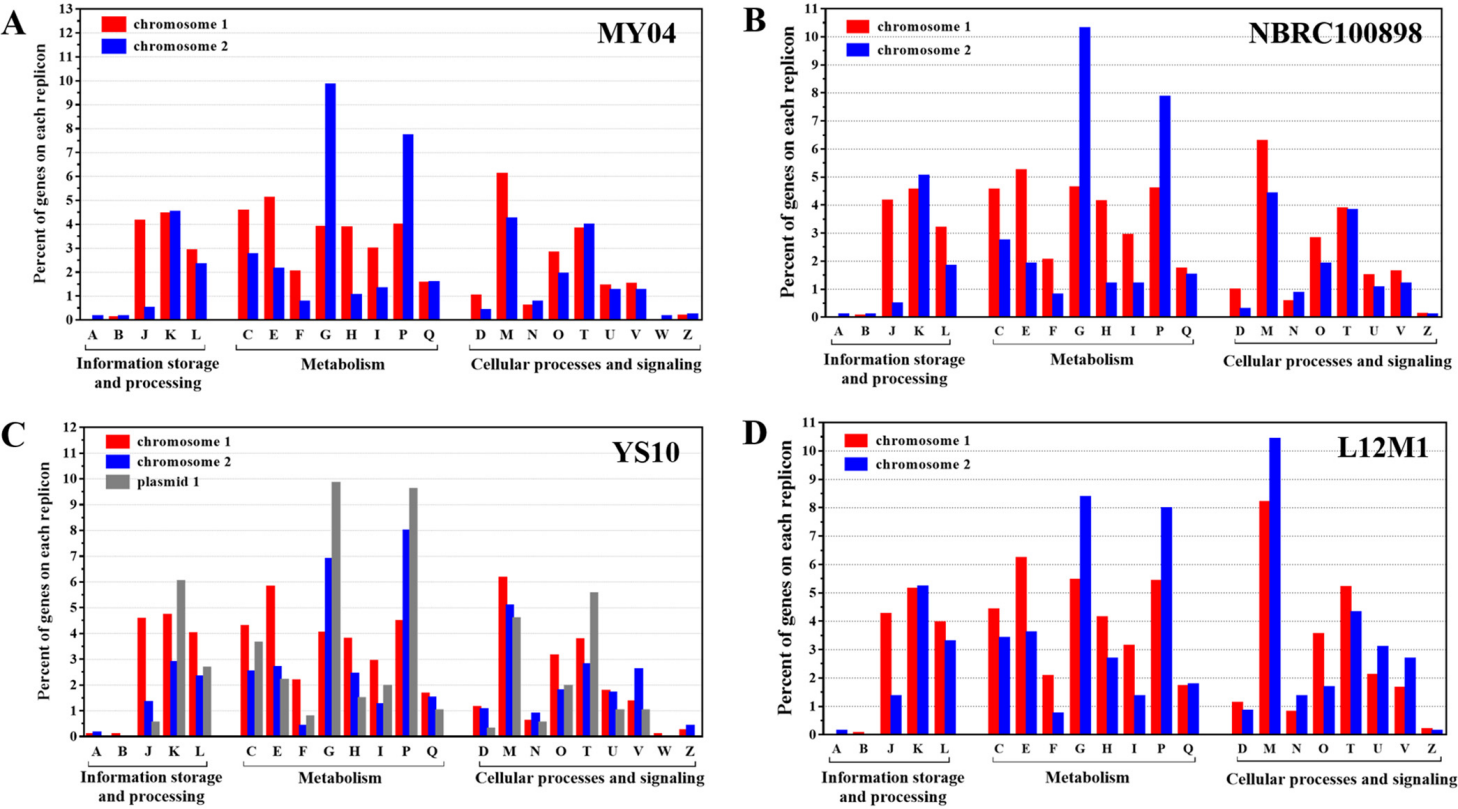
Functional distribution over each replicon based on the COG classifications of these genomes. (A) MY04. (B) NBRC 100898. (C) YS10. (D) L12M1. These COG features are divided into three broad categories: information storage and processing, cellular processes, and metabolism. The percentages of genes on each replicon belonging to each COG are presented, and the function of each COG is shown in Fig. 1. On chromosome II, the proportions of COG-P and COG-G are higher than those on chromosome I.

Based on the clusters of orthologous genes (COGs), we analyzed the functional preferences of proteins encoded by chromosomes in the MY04, YS10, NBRC 100898, and L12M1 genomes. The results showed that in MY04, the proportion of proteins encoded by chromosome I in translation, ribosomal structure, and biogenesis (COG-J) was very different from that of chromosome II, which corresponded to 4.06 and 0.41%, respectively. Analysis of the complete genome showed that there was no ribosomal assembly protein on chromosome II, which indicated that chromosome I was responsible for the translation process. In metabolic classifications, the proportions of proteins encoded by chromosome I in energy production and conversion (COG-C), amino acid transport and metabolism (COG-E), nucleotide transport and metabolism (COG-F), coenzyme transport and metabolism (COG-H), and lipid transport and metabolism (COG-I) were much higher than those of chromosome II. However, the proportions of carbohydrate transport and metabolism (COG-G) and inorganic ion transport and metabolism (COG-P) of chromosome I were much lower than those of chromosome II. The proteins annotated as COG-G and COG-P on chromosome II accounted for 9.76 and 7.65% of the total proteins, respectively, while the corresponding values of chromosome I were only 3.81 and 3.91%. There was no significant difference between the proteins encoded by the two chromosomes in cellular processes and signal classification (Fig. 2A). The results showed that chromosome I was responsible for core cell functions, such as cell replication, transcription, translation, and posttranslational modification, and had a preference for the transport and metabolism of amino acids, nucleic acids, coenzymes, and lipids. On the other hand, the proteins encoded by chromosome II had a significant preference for the transport of carbohydrates and inorganic ions.

In the genomes of NBRC 100898, YS10, and L12M1, the genes of COG-G and COG-P were also enriched on chromosome II. For example, in NBRC 100898, the proteins annotated as COG-G and COG-P on chromosome II accounted for 10.27 and 7.83% of the total proteins, respectively, while the corresponding values on chromosome I were only 4.60 and 4.55% (Fig. 2B). On chromosome II of YS10 and L12M1, the proportions of COG-P and COG-G were also higher than those on chromosome I (Fig. 2C and D). The transport and metabolism of carbohydrates may be related to the ability of the genus Flammeovirga to degrade many kinds of polysaccharides, while the transport and metabolism of inorganic ions may be related to the marine living environment of the strains. Therefore, we believed that large chromosome I of the genus Flammeovirga was the “core” chromosome, while small chromosome II affected the “lifestyle” of the strains.

### The second chromosomes of the genus Flammeovirga were enriched in glycoside hydrolases.

**FIG 3:**
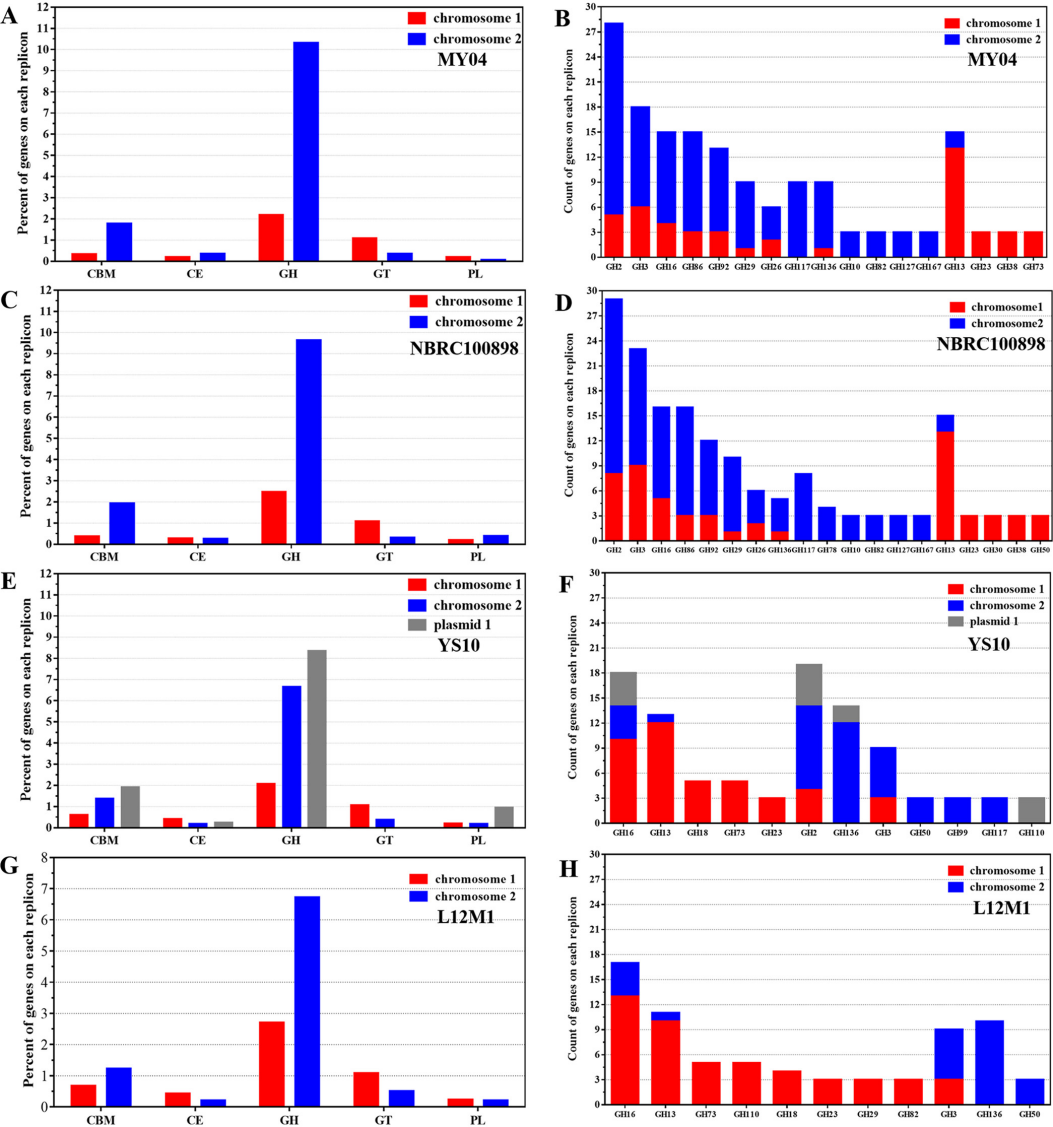
Preference of CAZy on different replicons of these genomes. (A) MY04. (C) NBRC 100898. (E) YS10. (G) L12M1. Carbohydrate-related genes in the carbohydrate-active enzyme (CAZy) database distribution over replicons. These genes are members of the carbohydrate-binding module family (CBM), glycoside hydrolase family (GH), glycosyl transferase family (GT), polysaccharide lyase family (PL), and carbohydrate esterase family (CE). The percentage of genes on each chromosome belonging to each CAZy is presented. A high proportion of GHs is significantly enriched on chromosome II. (B) MY04. (D) NBRC 100898. (F) YS10. (H) L12M1. Families with large differences in the number of GH distributions over each replicon are shown. The preferences of different GH families on different chromosomes are indicated, and each strain had its own characteristics.

Furthermore, the effects of the two chromosomes on the ability of MY04, NBRC 100898, YS10, and L12M1 to degrade polysaccharides were compared in detail (Table 2). The results showed that 10.31% of the coding protein genes of MY04 chromosome II were annotated as glycoside hydrolases (GHs), while only 2.18% were annotated on chromosome I (Fig. 3A). Among them, GH2, GH3, GH10, GH16, GH26, GH29, GH82, GH86, GH92, GH117, GH127, GH136, and GH167 were significantly enriched on chromosome II, while GH13, GH23, GH38, and GH73 were significantly enriched on chromosome I (Fig. 3B). We found that the GH families on chromosome I were mainly lysozyme, peptidoglycan lyase, and chitinase, while GHs on chromosome II degraded other polysaccharides, such as xylan, carrageenan, mannan, glucan, and agarose.

**TABLE 2:** The count distribution of carbohydrate-active enzymes of Flammeovirga genomes on different chromosomes^*a*^

Strain	Type	CBM	CE	GH	GT	PL
MY04	Chromosome I	13	8	86	43	8
	Chromosome II	26	5	151	5	1
NBRC 100898	Chromosome I	15	11	99	43	8
	Chromosome II	30	4	150	5	6
YS10	Chromosome I	22	15	76	39	7
	Chromosome II	15	2	73	4	2
	Plasmid	8	1	35	0	4
L12M1	Chromosome I	27	17	108	43	9
	Chromosome II	12	2	66	5	2

a CBM, carbohydrate-binding module family; CE, carbohydrate esterase family; GH, glycoside hydrolase family; GT, glycosyl transferase family; PL, polysaccharide lyase family.

Similarly, in NBRC 100898, YS10, and L12M1, chromosome II was also enriched with a large number of GHs relative to chromosome I. For example, the proportions of GHs on chromosome I and chromosome II of NBRC 100898 were 2.47 and 9.63%, respectively (Fig. 3C). In YS10 and L12M1, the corresponding proportions were 2.07 and 6.66% (Fig. 3E) and 2.70 and 6.71% (Fig. 3G), respectively. In addition to the preferences of different GH families on different chromosomes, each strain had its own characteristics (Fig. 3D, F, and H).

Therefore, the ability of the genus Flammeovirga to degrade many kinds of polysaccharides was closely related to chromosome II. This suggested that chromosome II may widen the niche of the strain so that it can obtain nutrition in different carbon source environments to improve the living space of the strains.

### The second chromosomes of the genus Flammeovirga were enriched in horizontally transferred genes.

**FIG 4:**
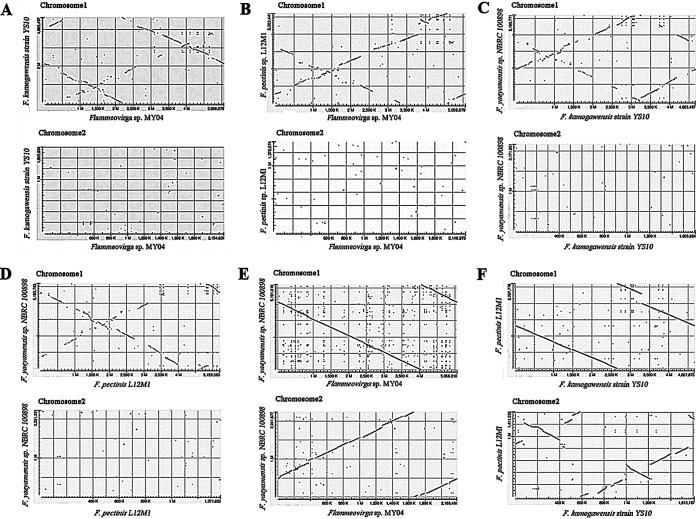
Genome synteny analysis of these genomes. (A) MY04 and YS10. (B) MY04 and L12M1. (C) YS10 and NBRC 100898. (D) L12M1 and NBRC 100898. (E) MY04 and NBRC 100898. (F) YS10 and L12M1. Genes in the genome with significant synteny show a clear line on the graph, while genes in the genome without synteny are scattered points in the graph. Except between MY04 and NBRC 100898 and between L12M1 and YS10, which are very closely related, the other genomes have synteny in chromosome I but not in chromosome II.

We also analyzed the synteny between the genomes of MY04, NBRC 100898, YS10, and L12M1. The results showed that synteny existed on chromosome I between MY04, YS10, and L12M1, while there was no synteny on chromosome II (Fig. 4A and B). There was synteny on chromosome I between NBRC 100898 and YS10 and L12M1, but there was no synteny on chromosome II (Fig. 4C and D). There was obvious synteny on chromosome I or II between MY04 and NBRC10089 and obvious synteny on chromosome I and synteny on chromosome II between YS10 and L12M1 as a consequence of closer phylogenetic relationships (Fig. 4E and F). This revealed that there was a higher mutation rate on chromosome II than on chromosome I in these genomes.

**FIG 5:**
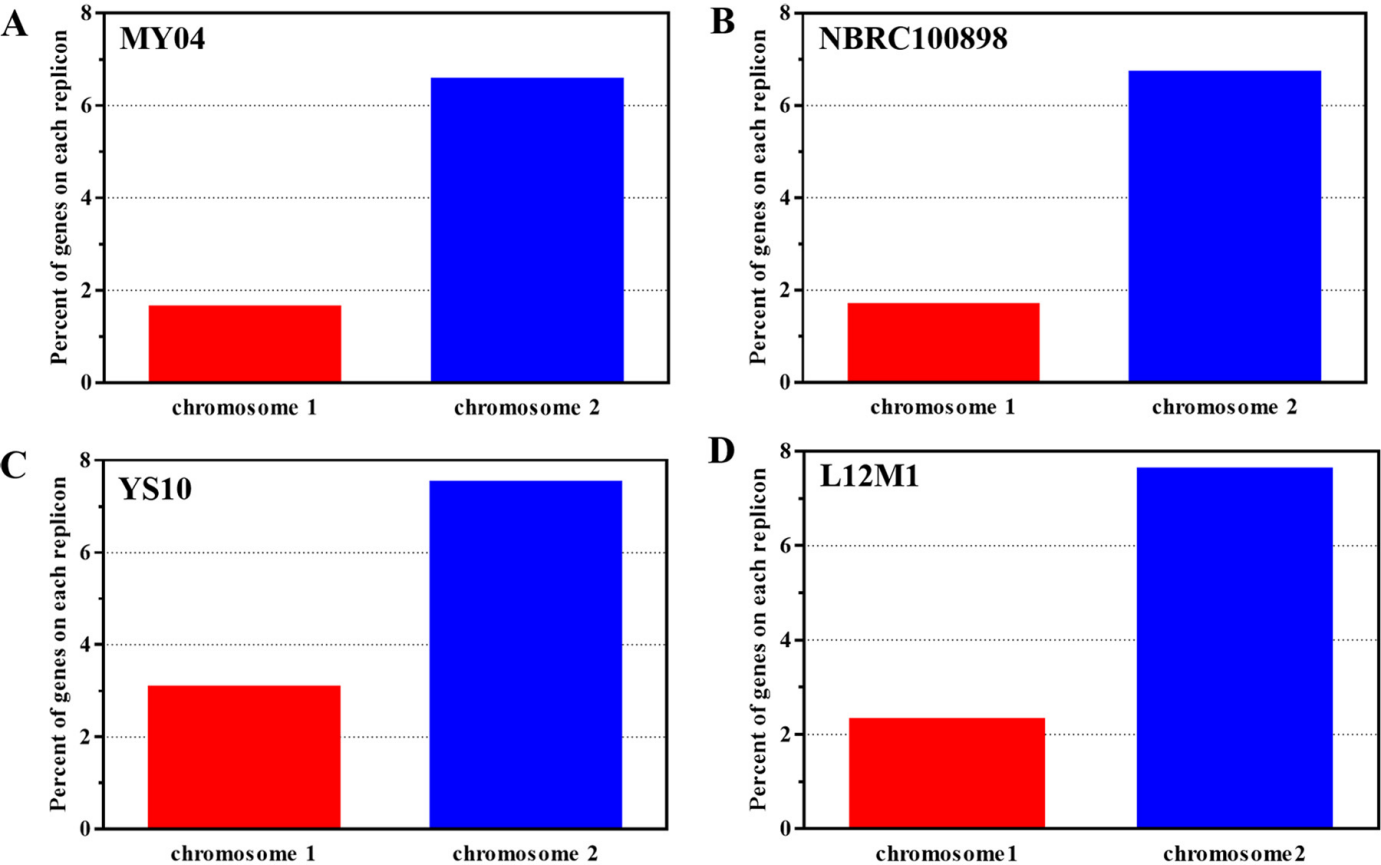
Distribution of HTG on different replicons. (A) MY04. (B) NBRC 100898. (C) YS10. (D) L12M1. The ordinate represents the percentage of the number of HTGs on each replicon to the total number of genes. Compared with chromosome I, chromosome II was rich in HTGs.

Therefore, we focused on the sources of genes in these genomes and analyzed their horizontal transfer genes (HTGs). The results showed that there were 66 genomic island (GI) genes on MY04 chromosome I, accounting for 1.62% of the total number of genes, while there were 97 GI genes on chromosome II, accounting for 6.55% of the total number of genes (Fig. 5A). In NBRC 100898, YS10, and L12M1, the HTGs on chromosome II were also higher than those on chromosome I (Fig. 5B to D). Therefore, compared with chromosome I, chromosome II was rich in HTGs.

At the same time, we used COG functional annotation to analyze the HTGs of the genus Flammeovirga. COG functions were divided into three clusters: cellular processes and signaling, information storage and processing, and metabolism. We found that 53% of HTGs on chromosome I were annotated, and only 25% of HTGs on chromosome II were annotated, which indicated that chromosome II obtained a large number of genes with unknown functions through horizontal gene transfer. We found that HTG functional annotation on chromosome I was enriched in metabolism, while chromosome II was enriched in cellular processes and signaling.

### The second chromosomes of the genus Flammeovirga are enriched in duplicated genes.

**FIG 6:**
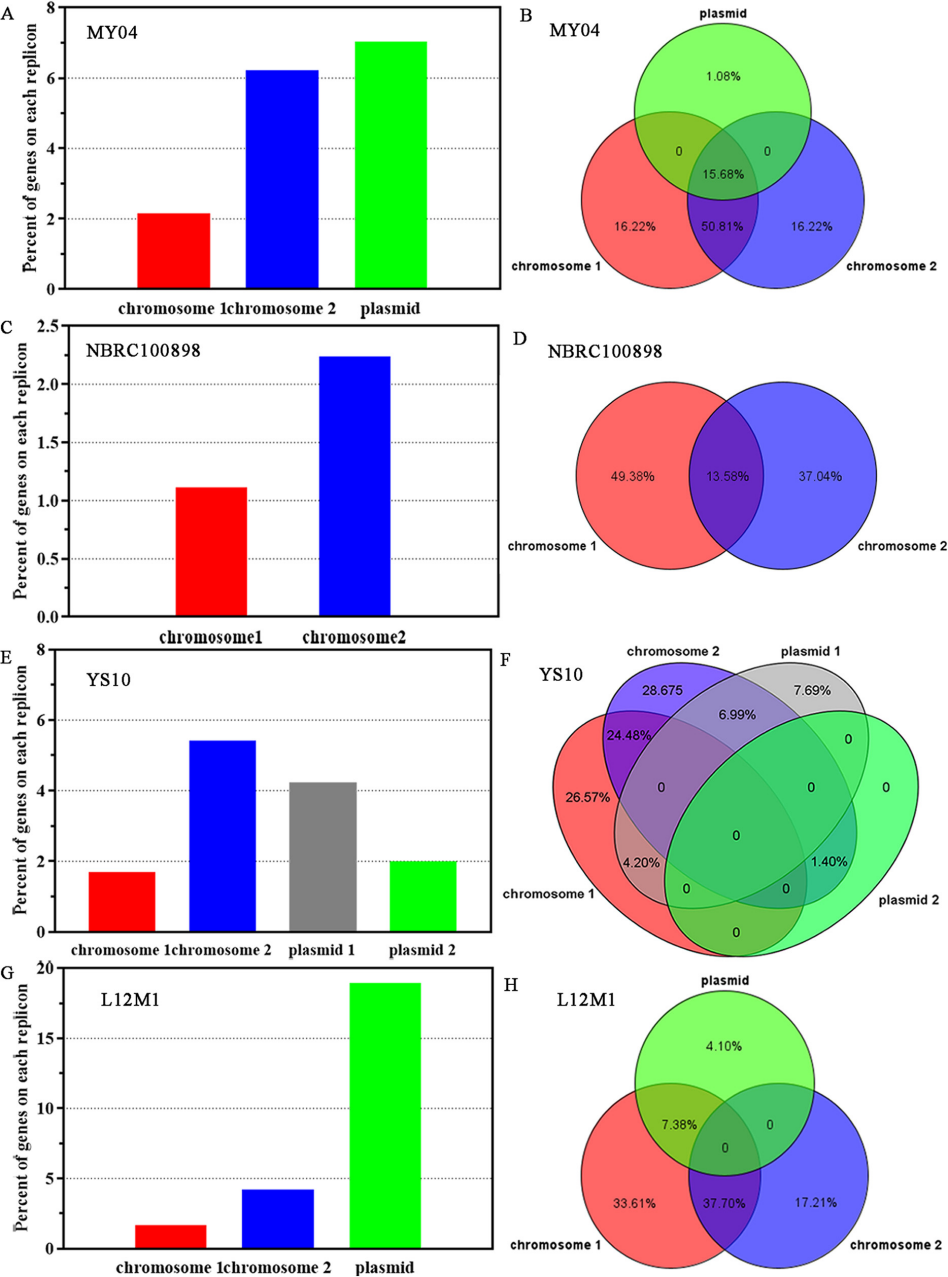
Distribution of duplicated genes on different replicons. (A) MY04. (C) NBRC 100898. (E) YS10. (G) L12M1. The ordinate represents the percentage of the number of duplicated genes on each replicon to the total number of genes. (B) MY04. (D) NBRC 100898. (F) YS10. (H) L12M1. The number represents the percentage of duplicated genes on different replicons and between replicons to the total number of duplicated genes. There was a higher proportion of duplicated genes on chromosome II than on chromosome I, and the distribution of these duplicated genes among different replicons showed differences in different strains.

In addition to HTGs, we also analyzed the sources of genes in the genus Flammeovirga from the perspective of duplicated genes. According to the standards that the gene sequence identity is more than 70% and that the mating coverage is more than 70%, we analyzed the duplicated genes in the genomes of MY04, NBRC 100898, YS10, and L12M1. The results showed that there were 185 genes in the MY04 genome with at least one duplicated gene (Fig. 6A). Among them, 6.21 and 7.04% of the genes were separately on chromosome II and plasmid with close duplicated, which were higher than 2.16% of chromosome I. For these gene pairs with high sequence identity, 16.22% were repeated only on chromosome I, 16.22% were repeated only on chromosome II, 1.08% were repeated on plasmids, 50.81% were repeated between chromosomes I and II, and 15.68% were repeated between chromosomes I and II and plasmids (Fig. 6B). Similar to MY04, there was a higher proportion of duplicated genes on chromosome II than on chromosome I in NBRC 100898, YS10, and L12M1 (Fig. 6C, E, and G). However, the distribution of these duplicated genes among different replicons showed differences in different strains (Fig. 6D, F, and H).

We also performed a functional cluster analysis of these duplicated genes. It was found that 65% of the duplicated genes on chromosome I were annotated, while only 53% of chromosome II were annotated, indicating that there was a large number of genes with unknown functions on chromosome II. Moreover, we found that the functional annotations of duplicated genes on chromosome I were enriched in cellular processes and signaling, while the functional annotations of duplicated genes on chromosome II were enriched in metabolism, which was exactly the opposite of the distribution of the functions of HTGs on the two chromosomes. This indicated that the abundant metabolic-related genes on chromosome II came from duplicated genes.

### Chromosome II of MY04 had lower selection pressure.

**FIG 7:**
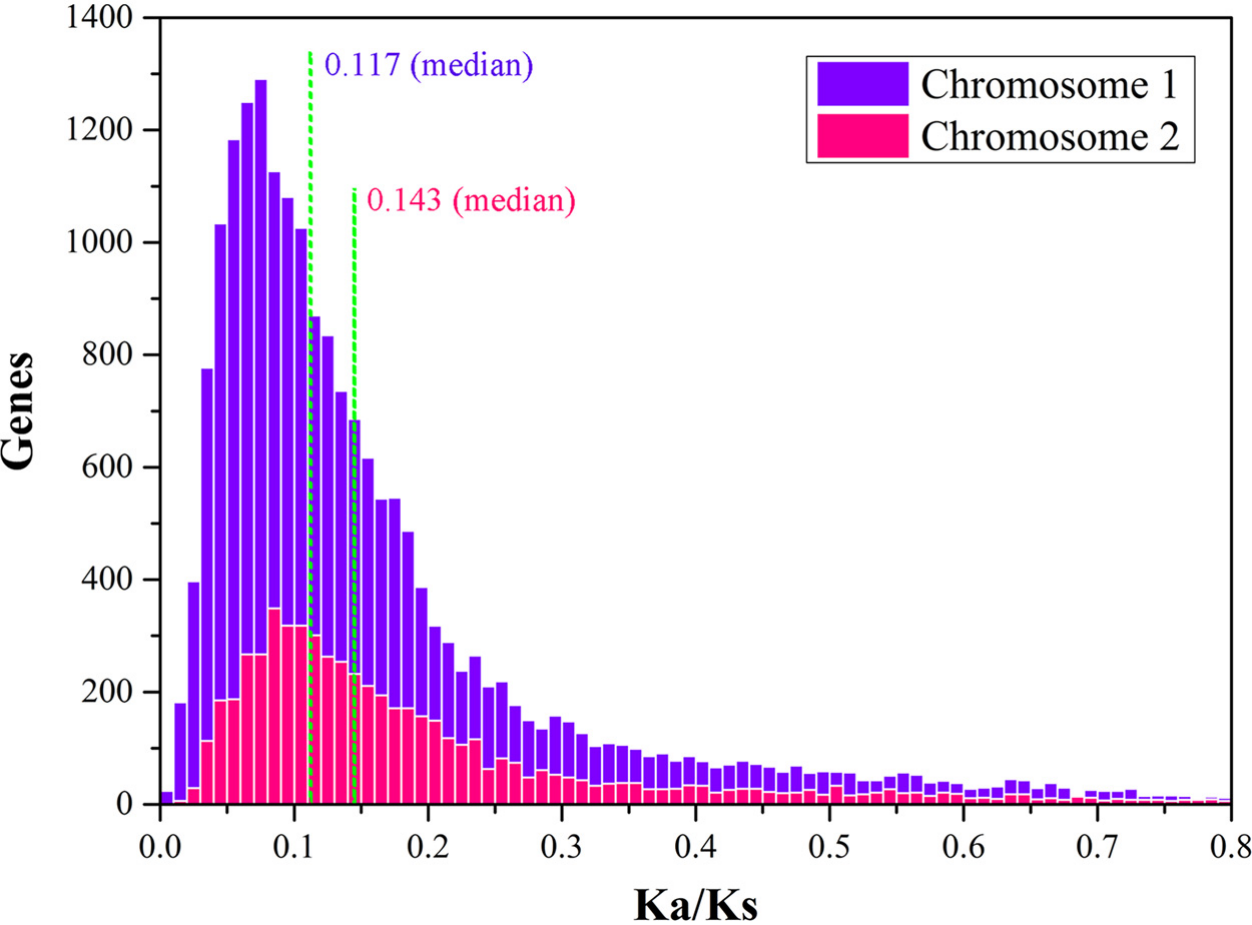
The ratio of nonsynonymous versus synonymous nucleotide substitutions (*K_a_*/*K_s_*) between orthologous pairs in Flammeovirga sp. strain MY04 and other strains, plotted conjointly for the two chromosomes. There is a significant trend toward more spread ratios (flattening of the distribution) from the large replicons to the smaller replicons. No positive selection could be detected overall for any pair of genes compared (i.e., *K_a_* > *K_s_*), and this trend was interpreted as a progressive relaxation of the selection pressure for amino acid substitution (the pressure of chromosome I > chromosome II).

To further test the difference in selection pressure acting on each replicon, we calculated the ratio of nonsynonymous substitution to synonymous substitution (*K_a_*/*K_s_*) between MY04 and the lineal homologous gene pairs of other strains. The *K_a_*/*K_s_* value was calculated by the MLWL and MLPB models, and the lower the value was, the stronger the purification selection was. The results showed that the median *K_a_*/*K_s_* of the chromosome I gene was 0.117, that of the chromosome II gene was 0.143, and that of the plasmid gene was 0.198 (Fig. 7). This suggested that the smaller chromosome II and plasmids were under more relaxed selection pressure than the larger chromosome I, which may adapt and drive their plasticity.

## DISCUSSION

With the development and improvement of next-generation genome-sequencing technology, multiple replicon systems have been found in an increasing number of bacterial strains, most of which are plant symbionts and human pathogens (1). As a result, increasing attention is focused on secondary replicons (e.g., the second chromosome), especially their origin, evolutionary history, function, and related mechanisms. Polysaccharide-degrading strains play an important role in the natural carbon cycle, and in recent decades, they have also been widely used in the development of carbohydrate active enzyme resources. However, little is known about their second chromosomes. Here, we report three complete sequenced genomes of strains MY04, NBRC10089, and YS10 of typical polysaccharide-degrading bacteria of the Flammeovirga genus and analyze the recently reported L12M1 genome at the same time. We found that these genomes contain not only primary chromosomes but also second chromosomes. The size of the second chromosome was much larger than the average size of conventional plasmids, and the GC contents were close to those of the primary chromosomes. The second chromosome encoded the core genes for bacterial growth. In addition, through comparative genome analysis, we inferred that the multireplicon system was the general genomic characteristic of the genus Flammeovirga.

There are two hypotheses about the formation mechanism of the second chromosome. One hypothesis is the division hypothesis (20, 24–27): the second chromosome is formed by the division of an ancestral chromosome. The other hypothesis is the plasmid hypothesis (28–32): the second chromosome evolves from plasmid expansion. According to the division hypothesis, the second chromosome should have high homology with the primary chromosomes and have the same core genes. For strains MY04, NBRC 100898, YS10, and L12M1, we found that the second chromosome had no obvious genome synteny with the primary chromosomes but quite different functional preferences. Furthermore, the primary chromosomes were predicted to be responsible for the basic survival of the bacterial host, while the second chromosomes were hypothesized to play a major role in lifestyle. The second chromosomes were enriched in duplicated genes and horizontal transfer genes, and the functions of the HTGs and duplicated genes of the two chromosomes were also different. In addition, there were replication and partitioning systems similar to those of plasmids (replicon III) on the second chromosome, the ParA-ParB-*parS* system, and RepA. The replication initiator protein of the second chromosome had very low homology with the primary chromosomes but had high homology with the replication initiator protein of the plasmid. The two chromosomes had different evolutionary characteristics: chromosome I was more conserved, and chromosome II had stronger plasticity. All of these results indicated that the second chromosome was derived from an ancestor plasmid. Therefore, we believe that the formation of the second chromosome of Flammeovirga might be closer to the hypothesis of evolution by plasmid expansion.

There are also four viewpoints on the benefits of the second chromosome to the strain: the first is to increase the size of bacterial chromosomes (33); the second is to increase the growth rate of cells (34); the third is to coordinate gene regulation (1); and the fourth is to adapt to the new living environment (5, 35, 36). For MY04, NBRC 100898, YS10, and L12M1, we found that these second chromosomes enriched a large number of genes encoding glycoside hydrolases, giving the strains the ability to degrade a variety of polysaccharides. The *K_a_*/*K_s_* value showed that the second chromosome had greater evolutionary plasticity than the primary chromosome under more relaxed selection pressure. All these results showed that in the process of evolution, the genus Flammeovirga might experience a harsh environment of oligonutrition and high salinity, forcing it to evolve a broader-spectrum oligosaccharide utilization ability to obtain nutrients and survive in the complex and changeable environment of the ocean. Therefore, we believe that the second chromosomes of the genus Flammeovirga promote the strain to adapt to the new living environment. In addition, studies on the distribution of multireplicon genomes in the entire bacterial phylogeny showed that the second chromosomes were universally present in a few genera. These species were able to survive in extreme environments due to their resistance to several pressures, such as UV radiation, metal ions, and aromatic compounds. These genera include Ralstonia, Deinococcus, and Cupriavidus (1). Studies have shown that one of the reasons that Deinococcus radiodurans is resistant to radiation is its efficient ability to repair damaged DNA. In addition to its efficient repair system, it also had chromosomal redundancy; that is, it had two chromosomes, and the extra genetic material protected the cell in two ways: when a large number of genomes existed, there were additional important site copies, thereby increasing the probability of cells surviving under radiation. Excess genetic information can also play a role in the repair of damaged fragments as reserved information (37). The bacteria of Flammeovirga all had a second chromosome. COG function cluster analysis found that there was a large number of genes with unknown functions and genes related to replication, recombination, and repair (COG L) on the second chromosome. These genes may promote bacteria to adapt to harsh environments.

In short, the second chromosomes of the genus Flammeovirga may come from an early plasmid, and then they are continuously amplified by many high-frequency horizontal gene transfer events, thus possessing the basic characteristics of the primary chromosome. The second chromosome expands the ability of the strain to degrade a variety of polysaccharides to obtain nutrition to improve its adaptation to the complex oligotrophic, open, and mobile marine environment. This study can be used as an example to provide a reference for peers to study the second chromosome or polysaccharide-degrading bacteria of the genus Flammeovirga.

## MATERIALS AND METHODS

### Source of the strain.

Flammeovirga sp. MY04 was isolated from coastal sediment in eastern China in 2004. F. yaeyamensis strain NBRC 100898 and F. kamogawensis strain YS10 were purchased from Marine Culture Collection of China.

### Genome sequencing and assembly.

Library construction and sequencing were performed at Shanghai Majorbio Biopharm Technology Co. Ltd. on Illumina HiSeq and Pacific Biosciences platforms. These strains were grown on agar plates in an atmosphere containing 5% CO_2_ at 37°C in fluid medium at 37°C with agitation. Genomic DNA sample(s) were isolated from the cell pellets with a bacteria DNA kit (Omega) according to the manufacturer’s instructions, and quality control was subsequently carried out on the purified DNA samples. Genomic DNA was quantified by using a TBS-380 fluorometer (Turner BioSystems Inc., Sunnyvale, CA). A highly qualified DNA sample (optical density at 260/280 nm [OD_260/280_] = 1.8 to 2.0, >6 µg) was utilized for construction.

For Illumina paired-end sequencing of each strain, at least 3 μg genomic DNA was used for sequencing library construction. Paired-end libraries with insert sizes of ∼400 bp were prepared following Illumina’s standard genomic DNA library preparation procedure. Purified genomic DNA was sheared into smaller fragments with a desired size by Covaris, and blunt ends were generated by using T4 DNA polymerase. After adding an A base to the 3′ end of the blunt phosphorylated DNA fragments, adapters were ligated to the ends of the DNA fragments. The desired fragments can be purified through gel electrophoresis and then selectively enriched and amplified by PCR. The index tag could be introduced into the adapter at the PCR stage as appropriate, and we performed a library quality test. Finally, the qualified Illumina paired-end library was used for Illumina NovaSeq 6000 sequencing (150 bp*2, Shanghai BIOZERON Co., Ltd.).

For Pacific Biosciences sequencing, 20k insert whole-genome shotgun libraries were generated and sequenced on a Pacific Biosciences RS instrument using standard methods. An aliquot of 8 μg DNA was spun in a Covaris g-TUBE (Covaris, MA) at 6,000 rpm for 60 s using an Eppendorf 5424 centrifuge (Eppendorf, NY). DNA fragments were then purified, end-repaired, and ligated with SMR Tbell sequencing adapters following the manufacturer’s recommendations (Pacific Biosciences, CA). The resulting sequencing libraries were purified three times using 0.45× volumes of Agencourt AMPure XPbeads (Beckman Coulter Genomics, MA) following the manufacturer’s recommendations.

The raw paired-end reads were trimmed and quality controlled by Trimmomatic with parameters (SLIDINGWINDOW:4:15 MINLEN:75) (version 0.36). Clean data obtained by the above quality control processes were used for further analysis.

Each genome was sequenced using a combination of PacBio RS and Illumina sequencing platforms. The Illumina data were used to evaluate the complexity of the genome and correct the PacBio long reads. First, we used ABySS to perform genome assembly with multiple-kmer parameters and obtained the optimal results of the assembly. Second, canu was used to assemble the PacBio-corrected long reads. Finally, GapCloser software was subsequently applied to fill up the remaining local inner gaps and correct the single base polymorphism for the final assembly results.

### Gene identification.

Gene annotation was added by the NCBI Prokaryotic Genome Annotation Pipeline (38, 39). The annotation method was the best-placed reference protein set and GeneMarkS+ (40). The annotated features included genes, coding sequences (CDSs), rRNAs, tRNAs, noncoding RNAs (ncRNAs), and repeat regions. The genome overview was created by BRIG to show annotation information (41).

### Functional gene analysis.

The COG functions of the Flammeovirga sp. strain MY04, F. yaeyamensis strain NBRC 100898, F. kamogawensis strain YS10 and F. pectinis strain L12M1 protein sequences were annotated by eggNOG-mapper v2 (e value ≥ 0.001, alignment coverage ≥ 20%) (42). The carbohydrate-active enzyme domains of these strains’ protein sequences were annotated by dbCAN-HMMdbv (43, 44).

### Duplicated genes and HTG analysis.

The gene sequences of Flammeovirga sp. strain MY04, F. yaeyamensis strain NBRC 100898, F. kamogawensis strain YS10, and F. pectinis strain L12M1 were subjected to self-repeat alignment using local BLAST (blast-2.7.1+) (e value threshold of 0.001) (45). Genes with alignment coverage and identity >70% were considered duplicated genes. Using IslandViewer 4 for HTG prediction, IslandViewer is a computational tool that integrates four different genomic island prediction methods: IslandPick, IslandPath-DIMOB, SIGI-HMM, and Islander (46).

### Evolutionary analysis.

CVTree constructs whole-genome-based phylogenetic trees without sequence alignment by using a composition vector (CV) approach (47). A phylogenetic tree was used to analyze the phylogenetic relationships of 12 strains (Flammeovirga sp. MY04, F. kamogawensis strain DSM 26273, F. yaeyamensis strain NBRC 100898, Flammeovirga pacifica WPAGA1, Flammeovirga sp. SR4, Flammeovirga sp. OC4, Flammeovirga sp. EKP202, Flammeovirga sp. SJP92, Flammeovirga aprica strain JL-4, F. kamogawensis strain YS10, F. kamogawensis strain DSM 26273, and F. pectinis) strain L12M1. A sequence alignment of the nucleic acid sequences based on their codons was established with TranslatorX and MAFFT (48). The *K_a_*/*K_s_* values among orthologous genes were calculated using KaKs_Calculator (49) with the MLWL and MLPB models (50).

### Data availability.

The complete genomes of Flammeovirga sp. MY04 (RefSeq Assembly Acceptance GCF_001682195.2), F. yaeyamensis strain NBRC 100898 (RefSeq Assembly Acceptance GCF_018736045.1), and F. kamogawensis strain YS10 (RefSeq Assembly Acceptance GCF_018736065.1) were released from the NCBI database. The main data supporting the findings of this study are available within the article. All other data supporting the findings of this study are available from the corresponding authors upon reasonable request.
